# Between Mosses and Microbes: Stable Isotopes ( ^15^N/ ^14^N, ^13^C/ ^12^C) Clarify the Trophic Niches of Oribatid Mites on a High‐Arctic Permafrost Archipelago (Svalbard, Norway)

**DOI:** 10.1002/ece3.74370

**Published:** 2026-09-21

**Authors:** Mark Maraun, Rasmus Clausen Kaiser, Stephen J. Coulson, Xue Pan, Stefan Scheu, Anna Seniczak

**Affiliations:** ^1^ JFB Institute of Zoology and Anthropology, Faculty of Biology and Psychology University of Göttingen Göttingen Germany; ^2^ University Centre in Svalbard Longyearbyen Norway; ^3^ College of Ecology and Environment Nanjing Forestry University Nanjing China; ^4^ Faculty of Applied Ecology, Agricultural Sciences and Biotechnology University of Inland Norway Elverum Norway

**Keywords:** oribatid mites, stable isotopes, Svalbard tundra, trophic niches

## Abstract

Tundra biomes are strongly affected by globally changing temperatures. Rising temperatures will increase soil biota activity and result in higher CO_2_ release from tundra soils and ‘greening’ of this biome, which in turn will affect carbon cycling and soil food webs. It is therefore important to better understand those high‐latitude soil food webs in tundra biomes. Here, we investigated adult and juvenile soil living oribatid mites (Acari) in eight different microhabitats (i.e., patches of vegetation from *Bistorta vivipara* , *Carex* sp./*Luzula* sp., *Cassiope tetragona* , *Dryas octopetala* , *Salix* spp. and *Saxifraga* spp., mosses and grasses) using stable isotope ratios (^15^N/^14^N, ^13^C/^12^C) in the Svalbard archipelago, a challenging habitat for soil biota with permafrost soils being entirely frozen for about 9 months of the year. Ten oribatid mite taxa were sufficiently abundant for stable isotope analyses, that is, *Camisia dictyna* , *Ceratoppia sphaerica* , *Diapterobates notatus* , *Dissorhina ornata* , *Fuscozetes setiger*, *Mycobates sarekensis* , Oppiidae, *Oribatula tibialis* , *Scutozetes arcticus* and *Tectocepheus sarekensis*. Four trophic groups of oribatid mites were identified (phytophagous—likely feeding on mosses, secondary decomposers, predators/scavengers and lichen feeders); primary decomposers were missing, pointing to low litter quality. The “detrital shift” in Δ^13^C was generally low pointing to the dominance of young carbon resources including mosses. Most oribatid mite taxa ( *Camisia dictyna* , *Ceratoppia sphaerica* , *Diapterobates notatus* , *Fuscozetes setiger, Mycobates sarekensis* and *Scutozetes arcticus*) were boreal/tundra habitat specialists. Juveniles and adults typically occupied the same trophic niche despite smaller body size and mouthparts in juveniles than in adults. Oribatid mite trophic niches did not differ between microhabitats indicating that they are not specifically adapted to certain plant species or microhabitats. Overall, the stable trophic ecology of oribatid mites across microhabitats allows predictions of Arctic soil food web changes under continued warming.

## Graphical abstract

Using stable isotopes (^15^N, ^13^C) we identified four trophic groups of oribatid mites (phytophagous, secondary decomposers, predators/scavengers and lichen feeders) on Svalbard; primary decomposers were missing, pointing to low litter quality. Most oribatid mite taxa were boreal/tundra habitat specialists with strong sclerotization which likely improves coping with cold and dry habitats. Juveniles and adults occupied the same trophic niche despite smaller body size and mouthparts than in adults.

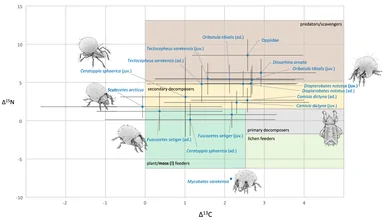

## 1 Introduction

Svalbard is an Arctic archipelago with a geological history spanning over 400 million years, but its current landscape is shaped by repeated glaciations during the Quaternary period (Yalcinkaya et al. 2022). The soils are predominantly nutrient‐poor and permafrost‐affected, classified as Cryosols, which limit deep rooting but preserve organic carbon over long timescales (Kimble 2004). Vegetation consists mainly of mosses, lichens, grasses and dwarf shrubs, adapted to extreme cold and a short growing season (Jónsdóttir 2005; Szymanski et al. 2022). Litter C‐to‐N ratios often exceed 35, indicating slow decomposition and nitrogen limitation in these cold environments (Hagedorn et al. 2025). Despite the limited biomass of plants, tundra ecosystems play a critical role in the Arctic carbon cycle: permafrost soils store large amounts of organic carbon, and warming‐induced thaw can release greenhouse gases, resulting in feedbacks that accelerate climate change (Schuur et al. 2015; Van den Broek et al. 2025). Soil microarthropods are key regulators of decomposition and nutrient cycling, and their activity may influence carbon release under future warming. However, their ecology in tundra soils has been little studied (Coulson et al. 2003).

The tundra of Svalbard forms a complex mosaic of microhabitats that influence below‐ground processes. The herb species *Bistorta vivipara* , mosses and *Carex/Luzula* species dominate moist, nutrient‐rich patches, creating dense tussocks that enhance soil moisture retention and organic matter accumulation. Dwarf shrubs such as *Cassiope tetragona* and *Dryas octopetala* occupy well‐drained ridges and gravelly slopes, moderating microclimatic extremes and stabilizing soils. Cryptogam species and low‐growing vascular plants like *Salix* spp. and *Saxifraga* spp. colonize snowbeds, crevices and patterned ground, providing localized refuges of high humidity and thermal buffering (Elvebakk 1997; Jónsdóttir 2005). These plant microhabitats likely structure soil microarthropod communities (Seniczak et al. 2014). By regulating litter breakdown and organic carbon turnover, microarthropods mediate the release from or retention of carbon in soil, making their interactions with plant microhabitats critical for predicting Arctic carbon fluxes under warming (Crowther et al. 2015). However, the importance of microhabitats for soil animal trophic ecology is little studied.

Soil animal communities in Svalbard are dominated by microarthropods, especially oribatid mites (Oribatida) and springtails (Collembola), which are key regulators of decomposition and nutrient cycling in the harsh Arctic environment (Zmudczynska et al. 2012; Seniczak et al. 2014; Gwiazdowicz et al. 2020). In particular, oribatid mites are abundant (Seniczak et al. 2026) and functionally diverse, feeding on detritus, algae, lichens, fungi, and bacteria (Magilton et al. 2019; Maraun and Scheu 2000; Maraun et al. 2023). These tiny organisms inhabit moss mats, litter layers, and the upper soil horizons, exploiting microhabitats buffered from extreme cold and desiccation.

Stable isotope analysis using ^15^N/^14^N and ^13^C/^12^C ratios provides a powerful tool to determine trophic niches of animals in complex communities (Perkins et al. 2014; Potapov et al. 2019). δ^15^N values indicate the trophic level of consumers, while δ^13^C values indicate the source of carbon, distinguishing between plant litter and microbially processed detritus (Peterson and Fry 1987; Post 2002; Potapov et al. 2019). The method therefore may be particularly suited for clarifying the food resources, trophic structure, and functional role of small tundra soil invertebrates such as oribatid mites.

We used stable isotope analysis of ^15^N/^14^N and ^13^C/^12^C ratios of adult and juvenile oribatid mites and their potential food resources (litter from different microhabitats) on Spitsbergen, Svalbard. In a first descriptive part of our study we investigated if (1) similar to temperate ecosystems, oribatid mites on Svalbard comprise five trophic groups, that is, phytophagous/bryophagous taxa, lichen feeders, primary decomposers, secondary decomposers and predatory/scavenging taxa; and if (2) oribatid mites from Svalbard include mainly endemic Arctic taxa. Moreover, we tested the following hypotheses: (1) juveniles and adults of the same species feed on similar food resources despite their different body size; and (2) the trophic niche of oribatid mites varies little between microhabitats, indicating limited dependency on variations in plant species community.

## 2 Materials and Methods

### 2.1 Study Sites, Sampling, and Species Determination

Samples including vegetation and soil, each 10 cm × 10 cm and 5 cm deep, were cut using a knife at five sites in Ebbadalen in Svalbard (Norway) on July 7 2023 (Table 1). Samples were taken from the following eight microhabitats at the five sampling sites in which a particular plant species (e.g., *Dryas octopetala* ) was highly dominant—if present: patches of (1) *Bistorta vivipara* (overall: 2 occurrences), (2) *Carex* sp./*Luzula* sp. (3 occurrences), (3) *Cassiope tetragona* (3 occurrences), (4) *Dryas octopetala* (15 occurrences), (5) *Salix* spp. (8 occurrences), (6) *Saxifraga* spp. (10 occurrences), (7) grasses (6 occurrences) and (8) mosses (13 occurrences) (Table 1). In samples dominated by mosses or grasses, the plants were not identified to species level; therefore, a single sample may have contained several moss species or several grass species. At each site, three subsamples (=sample ID) were taken from dominant plant microhabitats. Samples were kept cool (6°C) at the University Centre in Svalbard (UNIS) until being taken to the University of Inland Norway where the oribatid mites were extracted using a Tullgren heat extractor for 10 days. Oribatid mite species which were relatively abundant in the samples and those which could be determined under the stereomicroscope were sorted and stored in 96% ethanol for subsequent stable isotope analysis. The following species were included: adults and juveniles of *Camisia dictyna* , *Ceratoppia sphaerica* , *Diapterobates notatus* , *Fuscozetes setiger*, *Tectocepheus sarekensis* and *Oribatula tibialis* , and adults of *Mycobates sarekensis* , Oppiidae, *Dissorhina ornata* and *Scutozetes arcticus*.

**TABLE 1:** Coordinates and microhabitats/plants sampled (including regional sample IDs) from the five sites in Ebbadalen on Svalbard. Three subsamples (=sample ID) were taken at each of the five study sites.

Sites	Sample ID	Coordinates	Microhabitats/plants sampled at the study sites
1	A	78°41′58.1″N 16°39′46.3″ E	*Bistorta*, *Cassiope*, *Dryas*, *Saxifraga*, grass
	B	78°41′57.3″N 16°39′42.4″ E	*Dryas*, *Saxifraga*, moss
	C	78°41′56.4″N 16°39′41.2″ E	*Dryas*, *Saxifraga*, moss
2	A	78°41′57.1″N 16°39′31.3″ E	*Dryas*, *Saxifraga*, moss
	B	78°41′56.7″N 16°39′31.3″ E	*Dryas*, *Saxifraga*, moss
	C	78°41′56.1″N 16°39′31.9″ E	*Dryas*, *Salix*, *Saxifraga*, grass, moss
3	A	78°41′57.8″N 16°39′18.7″ E	*Dryas*, *Salix*, *Saxifraga*, grass, moss
	B	78°41′56.8″N 16°39′18.9″ E	*Dryas*, *Salix*, *Saxifraga*, grass, moss
	C	78°41′56.2″N 16°39′18.8″ E	*Bistorta*, *Dryas*, *Salix*, *Saxifraga*, grass, moss
4	A	78°41′57.3″N 16°38′47.4″ E	*Dryas*, moss
	B	78°41′50.0″N 16°38′47.9″ E	*Dryas*, moss
	C	78°41′58.6″N 16°38′48.7″ E	*Cassiope*, *Dryas*, *Salix*, grass
5	A	78°41′54.7″N 16°36′53.4″ E	*Carex*/*Luzula*, *Dryas*, *Salix*, moss
	B	78°41′55.4″N 16°36′48.3″ E	*Carex*/*Luzula*, *Dryas*, *Salix*, moss
	C	78°41′53.1″N 16°36′53.5″ E	*Carex*/*Luzula*, *Cassiope*, *Dryas*, *Salix*, *Saxifraga*, moss

### 2.2 Stable Isotope Analysis

Before stable isotope measurements, litter/plant and animal samples were dried at 60°C for 24 h. For each mite taxon and life‐stage, between 5 and 10 individuals (depending on body mass) were transferred into a single tin capsule. We merged oribatid mites from litter and soil as stable isotope ratios of Oribatida species are consistent across layers (Lu et al. 2022). Overall, we measured the following number of replicates per mite taxon/life‐stage: *Camisia dictyna* (adult/juvenile; 36/27), *Ceratoppia sphaerica* (adult/juvenile; 4/9), *Diapterobates notatus* (adult/juvenile; 44/36), *Dissorhina ornata* (adult; 19), *Fuscozetes setiger* (adult/juvenile; 17/2), *Mycobates sarekensis* (adult; 1), Oppiidae (adult; 45), *Oribatula tibialis* (adult/juvenile; 19/4), *Scutozetes arcticus* (adult; 5), *Tectocepheus velatus* (adult/juvenile; 41/26). Litter samples were grounded in a ball mill (Retsch Mixer Mill MM200, Haan, Germany) and 1–2 mg was weighed into tin capsules. Animal and litter samples were analyzed using a coupled system of an elemental analyzer (NA 1500, Carlo Erba, Milan, Italy) and a mass spectrometer (MAT 251, Finnigan, Bremen, Germany) adapted for the analysis of small sample sizes (Langel and Dyckmans 2014). Ratios of the heavy isotope to the light isotope (^13^C/^12^C, ^15^N/^14^N, denoted as R) were expressed in parts per thousand relative to the standard using the delta notation with δ^13^C or δ^15^N = (R_sample_/R_standard_ − 1) × 1000 (‰), with R_sample_ and R_standard_ being the respective target isotope ratios (^15^N/^14^N or ^13^C/^12^C). Vienna PD Belemnite limestone (V‐PDB) and atmospheric nitrogen were used as standard for ^13^C and ^15^N, respectively. Acetanilide (C_8_H_9_NO, Merck, Darmstadt) was used for internal calibration (for details see Reineking et al. 1993).

### 2.3 Data Analysis

For testing the first descriptive part of our study, we calibrated animal stable isotope values to litter from the respective microhabitats as baseline (denoted as Δ^13^C and Δ^15^N values) (Klarner et al. 2014; Potapov et al. 2019) and aggregated them into trophic groups according to Maraun et al. (2023). The grouping was done as follows: plant/moss feeders: Δ^13^C < 2.5, Δ^15^N < 1.7; primary decomposers: Δ^13^C 2.5–5.0, Δ^15^N −1.7–1.7; secondary decomposers/fungal feeders: Δ^13^C < 5, Δ^15^N 1.7–5.1; lichen feeders: Δ^13^C 2.5–5.0, Δ^15^N < −1.7, and predators: Δ^13^C < 5, Δ^15^N > 5.1 (Maraun et al. 2023). For testing the second descriptive part of our study, we investigated the world‐wide distribution of the studied oribatid mite species using data on the biogeography of oribatid mites (Xue Pan, unpubl. data; Global Oribatida Occurrence Data (GOOD)).

For testing our first hypothesis, we compared the calibrated Δ^13^C and Δ^15^N values of oribatid mite species for which we had juveniles and adults. Prior to statistical analyses, normality was assessed using the Shapiro–Wilk test, and homogeneity of variances was tested using Levene's test (with the car package). Because the assumptions of normality and homogeneity of variances were not met in some cases, differences in stable isotope values were analyzed using linear mixed‐effects models (LMMs) implemented in the nlme package. The comparison of juveniles and adults was included as a fixed effect, whereas sample ID was included as a random intercept to account for repeated measurements of the same individual. To account for heteroscedasticity, a residual variance structure was incorporated using the varIdent variance function. Models were fitted using restricted maximum likelihood (REML). Separate models were fitted for Δ^13^C and Δ^15^N values. The significance of the fixed effect was assessed using an analysis of variance (ANOVA) on the fitted mixed‐effects models. Model assumptions were evaluated by visual inspection of diagnostic plots. Residual normality was assessed using normal Q–Q plots and histograms of the model residuals, while homogeneity of variance was examined by plotting residuals against fitted values. Models were considered appropriate when residuals were approximately normally distributed and no systematic pattern or strong heteroscedasticity was observed in the residual diagnostic plots.

For testing the second hypothesis, we compared the uncalibrated δ^13^C and δ^15^N values of all oribatid mite species (including the juveniles) from the eight microhabitats. This analysis was done in the same way as the test of the first hypothesis (see above) using oribatid mite species as a fixed factor and sample ID as a random factor. All statistical analyses were performed in R (R Core Team, version 4.5.3).

## 3 Results

### 3.1 Trophic Structure

**FIGURE 1:**
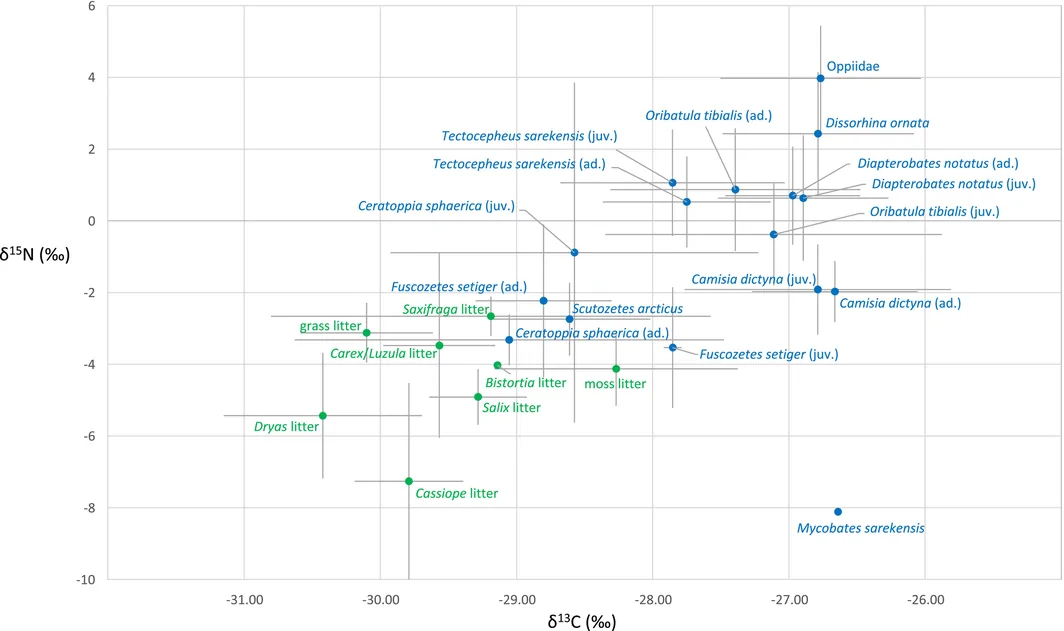
Non‐calibrated δ^15^N and δ^13^C values of oribatid mite species (adults and juveniles; in blue) and their potential food resources (organic material from eight microhabitats; in green) from the island of Spitsbergen, Svalbard. Points are means ± SD.

Non‐calibrated δ^15^N values of oribatid mites across the sampling sites spanned 12.1‰ from −8.1‰ in *Mycobates sarekensis* to 4‰ in Oppiidae; δ^13^C values spanned 2.4‰ from −29.1‰ in *Ceratoppia sphaerica* to −26.7‰ in *Camisia dictyna* (Figure 1).

**FIGURE 2:**
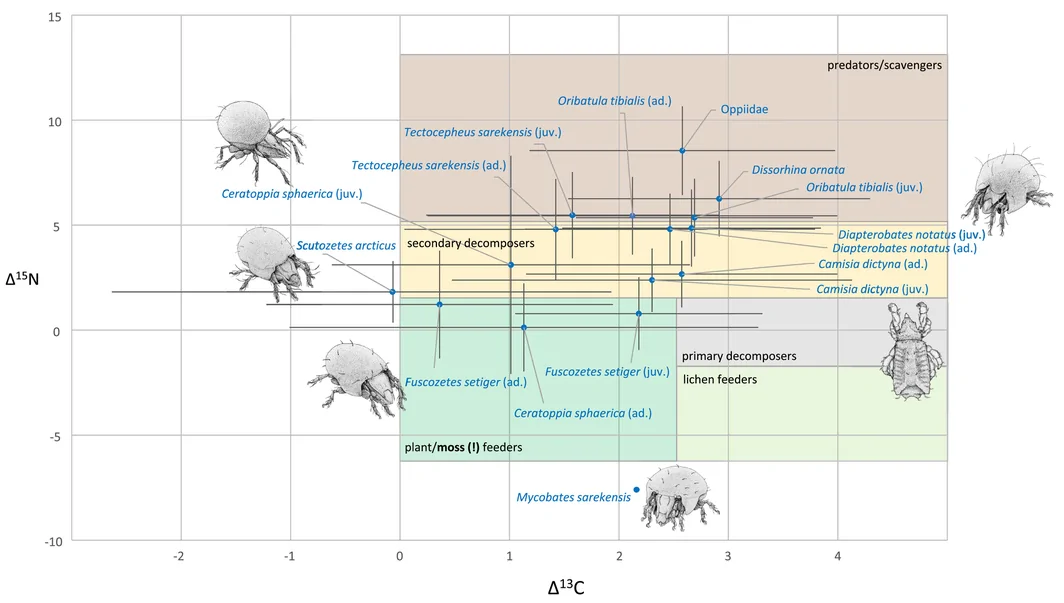
Stable isotope values (Δ^15^N, Δ^13^C) of oribatid mite species (adults and juveniles; in blue) from the island of Svalbard calibrated to litter of the respective study site. Oribatid mite species were ascribed to trophic groups as in Maraun et al. (2023) (in black). Points are means ± SD Animal drawings by Svenja Mayer.

Oribatid mites were ascribed to four trophic groups (Figure 2) (Maraun et al. 2023), that is, phytophagous taxa [ *Ceratoppia sphaerica* (adult), *Fuscozetes setiger* (juveniles and adults)], secondary decomposers [ *Ceratoppia sphaerica* (juveniles), *Diapterobates notatus* (juveniles and adults), *Camisia dictyna* (juvenile and adult), *Tectocepheus sarekensis* (adult)] and predators/scavengers [*Tectocepheus sarekensis* (juvenile), *Disshorina ornata*, *Oribatula tibialis* (juvenile and adult), Oppiidae]. The stable isotope signature of *Scutozetes arcticus* was close to plant feeders and secondary decomposers. The single measurement of *Mycobates sarekensis* points to this species feeding on lichens. No species was ascribed to primary decomposers.

### 3.2 Arctic Species and Their Biogeography

Oppiidae (including *Dissorhina ornata* ), *Tectocepheus sarekensis* and *Oribatula tibialis* are widely distributed cosmopolitan, euryoekous species, whereas the other species (*Diapteroabtes notatus*, *Ceratoppia sphaerica* , *Camisia dictyna* , *Fuscozetes setiger*, *Mycobates sarekensis* , *Scutozetes arcticus*) are habitat specialists that only occur in cold, Arctic, tundra biomes or at high altitudes. For details of their distribution areas see Figures S1–S9.

### 3.3 Trophic Niches of Juveniles and Adults

In five of the six species studied, neither Δ^15^N nor Δ^13^C values differed between juveniles and adults (ANOVA on LMM; *Camisia dictyna* : *F*_1,22_ = 1.23, *p* = 0.28 and *F*_1,22_ = 1.46, *p* = 0.24; *Ceratoppia sphaerica* : *F*_1,2_ = 2.17, *p* = 0.28 and *F*_1,2_ = 0.02, *p* = 0.89; *Diapterobates notatus* : *F*_1,33_ = 0.04, *p* = 0.85 and *F*_1,33_ = 2.57, *p* = 0.12; *Fuscozetes setiger*: *F*_1,1_ = 0.003, *p* = 0.97 and *F*_1,1_ = 4.94, *p* = 0.27; *Oribatula tibialis* : *F*_1,3_ = 0.08, *p* = 0.80 and *F*_1,3_ = 0.13, *p* = 0.74, for Δ^15^N and Δ^13^C values, respectively). In *Tectocepheus sarekensis*, Δ^13^C values also did not differ between juveniles and adults (*F*_1,25_ = 0.22, *p* = 0.65), but Δ^15^N values were higher in juveniles than in adults (*F*_1,27_ = 5.43, *p* = 0.028); however, the difference was small (0.68 Δ units; Figure 2). Model diagnostics indicated that residuals were approximately normally distributed and showed no strong deviation from homoscedasticity, supporting the adequacy of the fitted models (see Appendix S1).

### 3.4 Variation in Trophic Niches Between Microhabitats

Neither Δ^15^N (ANOVA on LMM: *F*_7,270_ = 0.93, *p* = 0.49) nor Δ^13^C values (*F*_7,270_ = 1.95, *p* = 0.06) of oribatid mite species significantly differed between microhabitats. Model diagnostics indicated that residuals were approximately normally distributed and showed no strong deviation from homoscedasticity, supporting the adequacy of the fitted models (see Appendix S1).

## 4 Discussion

We studied the trophic ecology of juvenile and adult oribatid mite species in eight different microhabitats in the tundra biome on Svalbard (Norway) using stable isotopes (^15^N/^14^N, ^13^C/^12^C). The overarching goal was to assess how the trophic roles of soil microarthropods vary across life stages and microhabitats in tundra environments.

### 4.1 Trophic Structure

Overall, oribatid mites spanned 12.1‰ in δ^15^N and 2.4‰ in δ^13^C which is similar to temperate and tropical ecosystems (Schneider et al. 2004; Illig et al. 2005; Krause et al. 2019). Conform to our first hypothesis, this indicates that, similar to temperate ecosystems, oribatid mites in Arctic Svalbard span a wide range of trophic groups. Although our study sites were close to the coast, δ^13^C values of oribatid mites indicate that they predominantly rely on autochthonous terrestrial resources and not on allochthonous resources of marine origin blown in by wind (Maberly et al. 1992).

However, in contrast to our first hypothesis, oribatid mites only grouped into four trophic groups, that is, plant/moss feeders, lichen feeders, secondary decomposers and predators. Primary decomposer and lichen feeders were rare or lacking; and also, no species was assigned to oribatid mites incorporating CaCO_3_ into their cuticle for protection against predators such as Phthiracaridae and species of the genus *Carabodes* (Maraun et al. 2023). This indicates that compared to temperate forest ecosystems the food web in the tundra biome of Svalbard is simplified (Zhemchuzhnikov et al. 2026). However, the range of δ^15^N values was similar to that of oribatid mite communities in temperate and tropical regions (Illig et al. 2005; Krause et al. 2019; Maraun et al. 2023), indicating that the trophic structure of the soil food web in Svalbard is generally comparable to those in temperate and tropical ecosystems.

Although a number of oribatid mite species from Svalbard are reported to feed on lichens (Konoreva et al. 2024), according to our dataset lichen feeders are rare, with only the single individual of *Mycobates sarekensis* ascribed to lichen feeders (see Pfingstl et al. (2025) for lichen feeding in the genus *Mycobates*). This rarity likely reflects that lichens are chemically well defended and generally of low food quality (Pöykkö et al. 2010). Primary decomposers feeding on organic material at early stages of decomposition were lacking entirely, generally pointing to low quality of litter resources at Svalbard. Low litter quality likely is related to low nitrogen availability due to the lack of nitrogen‐fixing plants in Svalbard, as well as the dominance of mosses and lichens which are both poor in nitrogen (Schimel and Bennett 2004). Similarly, in nutrient‐poor tropical soils oribatid mites functioning as primary decomposers are rare (Illig et al. 2005). The results generally point to microorganisms decomposing the low‐quality litter with animal detritivores predominantly feeding on microorganisms, presumably mainly fungi (Pollierer and Scheu 2021). Further, taxa incorporating CaCO_3_ into their cuticle such as Phthiracaridae were lacking. This is surprising since the parent rock at the study sites is based in large on limestone deposits (Mann et al. 1986), potentially, the uptake of calcium is constrained due to thick organic layers and permafrost soils. Overall, the results indicate that the oribatid mite community on Svalbard includes plant/moss feeders, secondary decomposers and predators reflecting the abundance of mosses (Wietrzyk‐Pełka et al. 2020), microorganisms and possibly nematode prey.

In contrast to temperate ecosystems, δ^13^C values of oribatid mites in Svalbard were little enriched compared to litter, that is, the detrital shift (Tiunov 2007; Potapov et al. 2019) was small. This may point to the differential use of litter compounds as basal resource with compounds depleted in ^13^C, such as lignin and waxes, being little used for microbial tissue formation and thereby for microbivore animal nutrition (Pollierer et al. 2009). Further, mosses as major resource of the food web in Svalbard do not contain lignin and only little cellulose (Maksimova et al. 2013), and this may explain the lack of a detrital shift in moss feeding taxa such as species of the genera *Fuscozetes*, *Ceratoppia* and probably also *Scutozetes*, however, this needs further testing.

### 4.2 Arctic Species

Supporting our assumption, the majority of the species from Svalbard were cold‐adapted Arctic species including *Camisia dictyna* , *Ceratoppia sphaerica* , *Diapterobates notatus* , *Fuscozetes setiger*, *Mycobates sarekensis* , and *Scutozetes arcticus*. *Diapterobates notatus* is the most often collected oribatid mite species in Svalbard (Seniczak et al. 2020) and can reach densities of up to 6000 specimens per m^2^ (Seniczak et al. 2017). *Scutozetes arcticus* is known only from the Arctic and recently has been reported from Svalbard (Seniczak et al. 2025). Many of the species (and their close relatives living in temperate regions) live on surfaces such as the bark of trees as lichen, moss, or algal feeders. However, the species in Svalbard also include cosmopolitan, ubiquitous species such as *Tectocepheus sarekensis*, Oppiidae (including *Dissorhina ornata* ), and *Oribatula tibialis* . Interestingly, these species are high up in the food web, whereas the cold‐adapted specialists occupy lower positions. Presumably, the cosmopolitan generalist species feed opportunistically on high‐quality resources including animal prey, such as nematodes, and live deeper in soil requiring less protection against desiccation.

Notably, typical primary decomposer taxa abundant in temperate forest soils were rare or lacking and this applies in particular to Desmonomata species of the genera *Nanhermannia*, *Nothrus*, and *Platynothrus*. This supports the conclusion that litter quality at Svalbard is generally poor, presumably related to the nutrient‐poor permafrost soils, limiting activity and survival of primary decomposer taxa.

### 4.3 Trophic Niches of Juveniles and Adults

The trophic position of five of the six studied oribatid mite species did not differ between adults and juveniles, generally supporting our third hypothesis. However, the trophic position of juveniles of *Tectocepheus sarekensis* was somewhat higher than that of adults. Considering the generally high trophic position of *Tectocepheus sarekensis* indicating that they live on animal prey, the results may indicate that the small body size of juveniles facilitates access to small prey such as nematodes. However, generally, the results suggest that the mouthparts of juveniles are sufficiently sclerotized to feed on the same resources as the adults. This supports earlier findings on *Nothrus* species that ^15^N/^14^N ratios of juveniles and adults do not differ significantly (Scheu and Falca 2000; Schneider et al. 2004). Generally, the data imply that juveniles and adults of oribatid mites may compete for the same food resources, and the similar trophic niche of both may explain why juveniles of oribatid mites are comparatively big.

### 4.4 The (Limited) Importance of Microhabitats

Neither Δ^15^N nor Δ^13^C values of oribatid mites differed significantly between the eight microhabitats studied supporting our fourth hypothesis. The wide range of trophic niches and feeding groups indicates that the trophic niche of each species occurs in each of the microhabitats. In fact, the major food resources, that is, mosses, microorganisms and nematodes, are likely to be present at each of the microhabitats investigated. Overall, the findings support earlier studies which also found the trophic niche of oribatid mites to vary little with the composition of tree species in forests (Eissfeller et al. 2013; Chen et al. 2024) and support the conclusion that generally, belowground animal diversity is less responsive to variations in plant diversity than animals above the ground (Hooper et al. 2000; Scherber et al. 2010; Cameron et al. 2019; Geisen et al. 2019; Seniczak et al. 2026). Eventually, our data show that, although differences in plant species create distinct soil conditions that lead to variation in oribatid mite community composition (Coulson et al. 2003), the trophic ecology of oribatid mites remains similar across these microhabitats.

## 5 Conclusions

In summary, our results demonstrate that the oribatid mite food web on Svalbard largely resembles that of temperate and tropical ecosystems, albeit it is somewhat simplified, with primary decomposers as well as taxa incorporating CaCO_3_ into their cuticle being rare or lacking. Moreover, the similarity in trophic niches of juveniles and adults indicates consistent resource use across developmental stages, despite differences in body size. The lack of microhabitat specific species underlines that oribatid mites are not closely linked to plant communities. Overall, these findings highlight the ecological flexibility and adaptive strategies of oribatid mites in Arctic environments, contributing to the persistence of soil food web functioning under extreme climatic conditions.

## Author Contributions

**Mark Maraun:** conceptualization (equal), data curation (equal), formal analysis (equal), investigation (equal), methodology (equal), resources (equal), software (equal), supervision (equal), validation (equal), visualization (equal), writing – original draft (equal), writing – review and editing (equal). **Rasmus Clausen Kaiser:** conceptualization (equal), data curation (equal), formal analysis (equal), investigation (equal), methodology (equal), validation (equal), writing – review and editing (equal). **Stephen J. Coulson:** formal analysis (equal), methodology (equal), resources (equal), visualization (equal), writing – review and editing (equal). **Xue Pan:** formal analysis (equal), methodology (equal), validation (equal), visualization (equal), writing – review and editing (equal). **Stefan Scheu:** formal analysis (equal), validation (equal), writing – review and editing (equal). **Anna Seniczak:** conceptualization (equal), data curation (equal), formal analysis (equal), investigation (equal), methodology (equal), project administration (equal), resources (equal), validation (equal), writing – original draft (equal).

## Funding

The authors have nothing to report.

## Conflicts of Interest

The authors declare no conflicts of interest.

## Supporting information


**Figure S1:** Sampling sites (red dots) where *Dissorhina ornata* (Oudemans, 1900) has been collected until 2024.
**Figure S2:** Sampling sites (red dots) where *Oribatula tibialis* (Nicolet, 1855) has been collected until 2024.
**Figure S3:** Sampling sites (red dots) where *Tectocepheus sarekensis* Trägårdh, 1910 has been collected until 2024.
**Figure S4:** Sampling sites (red dots) where *Camisia dictyna* Colloff, 1993 has been collected until 2024.
**Figure S5:** Sampling sites (red dots) where *Ceratoppia sphaerica* (L. Koch, 1879) has been collected until 2024.
**Figure S6:** Sampling sites (red dots) where *Diapterobates notatus* (Thorell, 1871) has been collected until 2024.
**Figure S7:** Sampling sites (red dots) where *Mycobates sarekensis* (Trägårdh, 1910) has been collected until 2024.
**Figure S8:** Sampling sites (red dots) where *Fuscozetes setiger* (Trägårdh, 1910) has been collected until 2024.
**Figure S9:** Sampling sites (red dots) where *Scutozetes arcticus* Ermilov & Makarova, 2021 has been collected until 2024.

**Data S1:** ece374370‐sup‐0002‐supinfo.pptx.

## Data Availability

Data are available in the DRYAD data repository (https://doi.org/10.5061/dryad.69p8cz9jv).
